# Probing High Affinity Sequences of DNA Aptamer against VEGF_165_

**DOI:** 10.1371/journal.pone.0031196

**Published:** 2012-02-16

**Authors:** Harleen Kaur, Lin-Yue Lanry Yung

**Affiliations:** Department of Chemical and Biomolecular Engineering, National University of Singapore, Singapore, Singapore; Ottawa Hospital Research Institute, Canada

## Abstract

Vascular endothelial growth factor (VEGF_165_) is a potent angiogenic mitogen commonly overexpressed in cancerous cells. It contains two main binding domains, the receptor-binding domain (RBD) and the heparin-binding domain (HBD). This study attempted to identify the specific sequences of the VEa5 DNA aptamer that exhibit high binding affinity towards the VEGF_165_ protein by truncating the original VEa5 aptamer into different segments. Using surface plasmon resonance (SPR) spectroscopy for binding affinity analysis, one of the truncated aptamers showed a >200-fold increase in the binding affinity for HBD. This truncated aptamer also exhibited high specificity to HBD with negligible binding affinity for VEGF_121_, an isoform of VEGF lacking HBD. Exposing colorectal cancer cells to the truncated aptamer sequence further confirmed the binding affinity and specificity of the aptamer to the target VEGF_165_ protein. Hence, our approach of aptamer truncation can potentially be useful in identifying high affinity aptamer sequences for the biological molecules and targeting them as antagonist for cancer cell detection.

## Introduction

Short single stranded nucleic acids referred to as aptamers are widely being explored as molecules of high affinity and specificity for binding a diverse array of target molecules ranging from high molecular weight proteins to small ions and nucleotides [1]–[3]. Aptamers contain functional moieties that fold into different secondary conformations, such as hairpin stem and loops, G-quadruplexes, bulges and pseudoknots, and they exhibit substantial impact on the conformational stability and target binding affinity of the aptamer. The non-immunogenic property of aptamer provides additional advantage over the prevalent antibodies and makes them a promising candidate for therapeutic and diagnostic application [4]. “Macugen” is the first FDA-approved aptamer-based therapeutic for treating the wet-form of age-related macular degeneration. The successful approval of this 27-mer RNA aptamer as therapeutic drug in 2004 has demonstrated the potential of aptamers as future therapeutics [5]. Currently, 8 aptamers are in various phases of clinical trials for treating different diseases [6]–[13]. These include NOX-E36 L-RNA aptamer against CCL2 ligand in Type 2 diabetes, G-quadruplex forming AS1411 DNA aptamer against nucleolin in acute myeloid leukemia, and phosphorothioate-modified ARC1779 DNA aptamer against von Willebrand factor (vWF) in carotid artery disease [7], [8], [10].

Aptamers are commonly screened and obtained by *in vitro* selection technique, also termed as systematic evolution of ligands by exponential enrichment (SELEX). SELEX starts with a random pool of oligonucleotide library incubated with the target molecule and involves continuous rounds of affinity and amplification steps to screen for the high affinity sequences [14], [15]. Different selection process has been used for isolation of high binding affinity aptamers in SELEX, such as nitrocellulose membrane filtration, surface plasmon resonance (SPR), capillary electrophoresis and bead-based methods [15]–[18]. Binding affinity and specificity are the crucial criteria for the therapeutic use of aptamers. Generally, not all nucleotide domains of the post-screened aptamer play an important role in target binding. The non-binding domain may actually interfere with the interaction between the aptamer and target protein by formation of complex secondary structures, and eventually prevents the binding domain to fold into the desired conformation for binding to the target [4]. This may result in reduction or complete loss of the binding affinity as well as higher synthesis cost. Therefore, identifying the high binding affinity domains in the post-screened aptamer is a key step to perform for producing potent aptamers with higher affinity/specificity for various biomedical applications.

Different strategies have been adopted to enhance the aptamer binding affinity for its target and to make them suitable for different biological applications. One of the commonly used strategies includes chemical modification of the aptamer structure at 5′- or 3′-terminus, nucleobase, sugar, and phosphate backbone. The modifications include (i) the addition of functional groups, such as amino (-NH_2_), fluoro (-F), *O*-methyl (-OCH_3_), locked nucleic acids (LNAs) or phosphorothioate linkages (PS-linkages) to make aptamers nuclease resistant, and (ii) conjugation with high molecular weight polyethylene glycol (PEG) to enhance *in vivo* circulating half-life [5], [19]–[26]. Apart from improving the stability of the aptamer, these modifications can improve the affinity of the aptamer in the cellular environment. Additionally, software algorithms have been used to deduce the binding domains by comparing different sequences as well as to predict the secondary structure [27], [28]. Strategies such as partial fragmentation, enzymatic footprinting, and recently microarray based binding sequence determination have also been employed for probing the high affinity binding sequences [13], [29], [30].

Vascular endothelial growth factor (VEGF) is a mitogenic protein secreted by both endothelial and tumor cells and induces physiological and pathological angiogenesis inside the body. Through alternate exon splicing of single human VEGF gene, several isoforms of this growth factor, including VEGF_165_, VEGF_121_, VEGF_189_ and VEGF_206_, are generated [31]. Of these, VEGF_165_ and VEGF_121_ are the predominant isoforms. VEGF_165_, which contains both heparin-binding domain (HBD) and receptor-binding domain (RBD), has been shown to be a more potent mitogen in inducing angiogenesis compared with VEGF_121_, which contains only RBD [32]–[34].

A previous work by Ikebukuro and co-workers has identified a 66mer DNA aptamer (VEa5) that binds to HBD of VEGF_165_ protein with K_d_ value of 130 nM [35]. Since increasing the binding affinity of this aptamer is important for further therapeutic development, they later adopted the dimerization method to reduce the K_d_ of VEa5 aptamer to 6 nM, which is 20 times better than the original monomeric VEa5 [36]. In the present study, we adopted a different strategy to improve the binding affinity of the VEa5 aptamer. We attempted to identify the high affinity binding sequences within the 66mer VEa5 by truncating its stem-loop regions and investigated the impact on the binding affinity against HBD of VEGF_165_ protein using surface plasmon resonance (SPR) spectroscopy. Our results demonstrated that the truncation of the stem-loop regions can locate the key binding domain in the aptamer and the truncated sequence exhibits substantially higher binding affinity compared with the entire VEa5 aptamer. *In vitro* binding study with colorectal cancer cells overexpressed with VEGF protein further confirmed the high binding affinity of the truncated aptamer.

## Materials and Methods

### Materials

The HPLC purified oligonucleotides (both unlabeled and fluorescent-labeled) were purchased from Sigma-Aldrich. The recombinant human carrier free VEGF_165_ (molecular weight of 38 kDa, pI = 8.25) and VEGF_121_ (molecular weight of 28 kDa, pI = 6.4) proteins were purchased from R & D systems. CM5 sensor chips were purchased from GE Healthcare for protein immobilization. 1-ethyl-3-[3-dimethylaminopropyl] carbodiimide hydrochloride (EDC), N-hydroxysuccinimide (NHS), and ethanolamine-HCl were purchased from Sigma-Aldrich. Sodium acetate (anhydrous) was purchased from Fluka. Human colorectal adenocarcinoma HT-29 cell line was a gift from Dr. Partha Roy's lab. Normal human fetal lung fibroblast MRC-5 cells were obtained from ATCC. Dulbecco's modified eagle's media (DMEM) media, RPMI-1640 and fetal bovine serum (FBS) was purchased from Caisson laboratories. Trypsin-EDTA and 1% penicillin/streptomycin mixture were purchased from PAN biotech. Phosphate buffer saline (PBS) buffer was purchased from 1^st^ Base. Tween-20 was purchased from USB Corporation.

### Binding affinity of truncated aptamers via surface plasmon resonance spectroscopy

**Figure 1:**
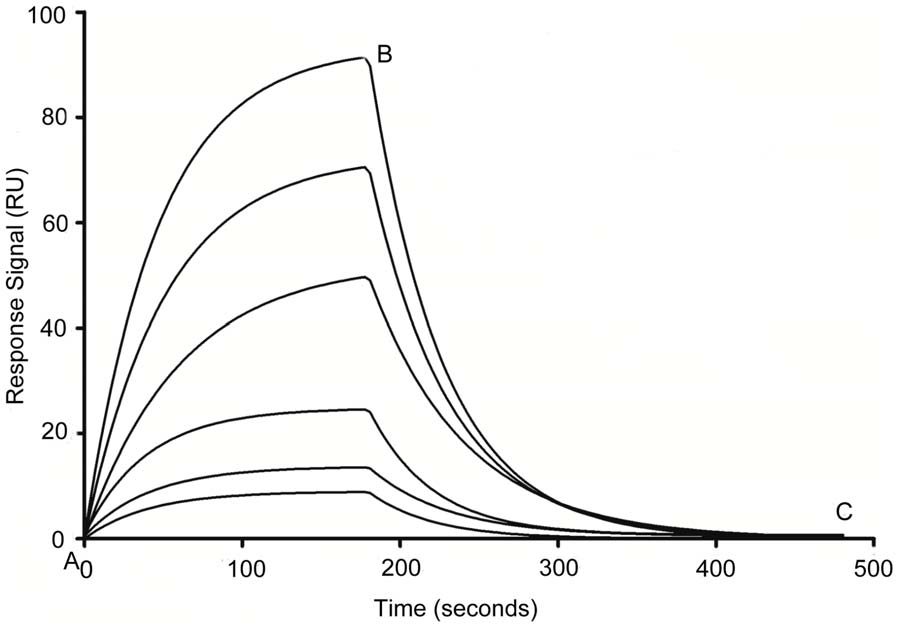
Typical SPR sensorgrams demonstrating interaction of aptamer with immobilized VEGF_165_ protein at different concentrations (bottom to top, 0.2 to 100 nM). Point A to B corresponds to the association phase and point B to C corresponds to the dissociation phase in all the sensorgrams. Shown here is the SL_2_-B aptamer (K_d_ = 0.50±0.32 nM).

To elucidate the role of stem-loop regions of the VEa5 aptamer in VEGF binding, the original sequence of the VEa5 was truncated. The corresponding binding affinity of truncated aptamers was investigated using surface plasmon resonance (SPR) spectroscopy, where VEGF_165_ and VEGF_121_ acted as ligands and were directly immobilized on the sensor chip. Briefly, the carboxylic group on the sensor chip was activated by standard amine coupling procedure using freshly prepared EDC/NHS. VEGF_165_ or VEGF_121_ (25 µg/ml) in acetate buffer (pH 6.0) was then injected into the sensor chip at flow rate 8 µl/min to reach ∼200RU immobilization level. The deactivation was done by ethanolamine-HCl to block unreacted carboxyl groups. The binding analysis was carried out with truncated aptamers at different concentrations (0.2 to 100 nM) using a BIAcore 2000 instrument (GE Healthcare). The running condition was set at 30 µl/min flow rate, 25°C, 3 min association time and 5 min dissociation time. PBS and 0.005% tween-20 solution mixture was used as the running buffer, and 50 mM NaOH as the regeneration buffer. All the buffers were filtered and degassed prior to each experiment. Blank surfaces were used for background subtraction. Upon injection of the truncated aptamers, sensorgrams recording the association/dissociation behavior of the VEGF-aptamer complex were collected. By varying the truncated aptamer concentration, a series of sensorgrams (Figure 1) were obtained and subsequently analyzed using the 1∶1 Langmuir model provided in the BIAevaluation software (version 4.1) to calculate the equilibrium dissociation constant K_d_. All SPR measurements were performed in triplicates.

### Cellular binding of truncated aptamer via flow cytometry analysis

The HT-29 colorectal cancer and MRC-5 human fetal lung fibroblast cells were plated at a seeding density of 10^5^ cells/ml in DMEM and RPMI-1640 media and were allowed to attach for 48 hours in humidified incubator containing 5% CO_2_, 1% O_2_ and 94% N_2_ (hypoxia) at 37°C. The hypoxia condition was maintained by culturing the cells in a sealed hypoxia chamber (Billups-Rothenberg). The cells were trypsinized for very short time and incubated with 5′-PE-texas red labeled truncated aptamer of different concentrations for 2 hours at 37°C in culture medium. The cells were then centrifuged for 5 min at 1500 rpm and re-suspended in PBS buffer for flow cytometry analysis immediately. Analysis was performed on a Beckman-Coulter CyAn ADP flow cytometer using 488 nm excitation and 613/20 nm emission filter. 20,000 events were collected for each sample. All the experiments for binding assay were repeated at least 3 times. Relative fluorescence was determined using SUMMIT V 4.3.02 software. For calculation of equilibrium dissociation (K_d_), the fluorescence intensity signal was plotted against fluorescently labeled aptamer concentration by fitting in equation Y = B_max_ X/(K_d_+X) using SigmaPlot software. Sequence specificity of the SL_2_-B aptamer was determined using a scrambled sequence. The K_d_ value of the SL_2_-B aptamer was calculated by subtracting the fluorescence intensity signal from the scrambled sequence.

Competitive aptamer binding assay was performed to determine the effect of unlabeled SL_2_-B aptamer on the binding capability of 5′-PE-texas red labeled truncated aptamer. HT-29 colorectal cancer cells were incubated with 20-fold excess concentration of unlabeled aptamer as competitor (10 nM) simultaneously with 5′-PE-texas red labeled aptamer (0.5 nM). All other experimental conditions and procedures were same as described for the flow cytometry analysis.

### Fluorescence microscopy imaging

The HT-29 cells and MRC-5 cells were seeded in 24-well plate in DMEM and RPMI-1640 media respectively. Cells were plated at a seeding density of 10^5^cells/ml in their individual media supplemented with FBS and penicillin/streptomycin mixture in the same hypoxic conditions as mentioned above. Subsequently, the cells were incubated with 5′-PE-texas red labeled truncated aptamer at 37°C for 2 hours and washed with PBS (pH = 7.4) three times to remove unbound aptamer. Sequence specificity of the truncated aptamer was determined using a scrambled sequence as control. Images of aptamer binding to cells were acquired using a Leica DMIL fluorescence microscope.

### Statistical analysis

Results from at least 3 independent experiments in flow cytometry experiment were analyzed using Student's t-test. p-value<0.05 was considered significant. Data are expressed as mean ± S.D.

## Results and Discussion

### Binding analysis of aptamer-VEGF complex by surface plasmon resonance (SPR)

**Figure 2:**
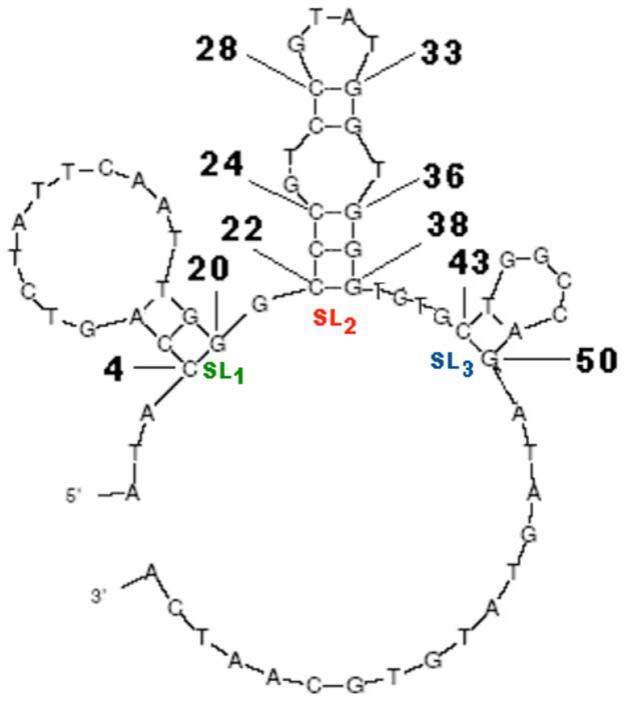
Schematic representation of the secondary structure of original VEa5 aptamer as predicted by the mfold program. Nucleotide 4–20 forms the stem-loop 1 (SL_1_) region, nucleotide 22–38 forms the stem-loop 2 (SL_2_) region, and nucleotide 43–50 forms stem-loop 3 (SL_3_) region. Nucleotide 28–33 forms internal loop 1 (IL_1_), and nucleotide 24–27+34–36 together forms internal loop 2 (IL_2_) within SL_2_ region.

The induction of the aptamer folding was done using predictions by the mfold software [37]. As shown in Figure 2, VEa5 displays complex hairpin stem-loop secondary structure with three stem-loop regions and several unpaired terminal nucleotides. Based on the SPR measurement, the original VEa5 aptamer exhibited a binding constant of K_d_ = 120 nM to the surface immobilized VEGF_165_ (Table 1). This value is very close to the K_d_ value of the VEa5 aptamer binding to the heparin-binding domain (HBD) of VEGF_165_ reported in the literature (K_d_ = 130 nM) [35].

**Table 1:** Different aptamer sequences along with their equilibrium dissociation constant (K_d_) values determined using surface plasmon resonance (SPR) spectroscopy.

Sequences of original and various truncated aptamers (5′------ 3′)*	K_d_
**VEa5 ATACCAGTCTATTCAATTGGGCCCGTCCGTATGGTGGGTGTGCTGGCCAGATAGTATGTGCAATCA **	120±1.8 nM
**SL_12_ ATACCAGTCTATTCAATTGGGCCCGTCCGTATGGTGGGTGTGCTGGCCAGATAGTATGTGCAATCA **	5±0.45 nM
**SL_1_ ATACCAGTCTATTCAATTGGGCCCGTCCGTATGGTGGGTGTGCTGGCCAGATAGTATGTGCAATCA **	No Binding
**SL_2_ ATACCAGTCTATTCAATTGGGCCCGTCCGTATGGTGGGTGTGCTGGCCAGATAGTATGTGCAATCA **	49±2.4 nM
**SL_2_-A ATACCAGTCTATTCAATTGGGCCCGTCCGTATGGTGGGTGTGCTGGCCAGATAGTATGTGCAATCA **	10±1.1 nM
**SL_2_-B ATACCAGTCTATTCAATTGGGCCCGTCCGTATGGTGGGTGTGCTGGCCAGATAGTATGTGCAATCA **	0.5±0.32 nM

*The underlined and bold section indicates the aptamer sequence and the non-bold grey section indicates the truncated sequence. In aptamer sequence terminology, the subscript number indicates the presence of the particular stem-loop region.

**Figure 3:**
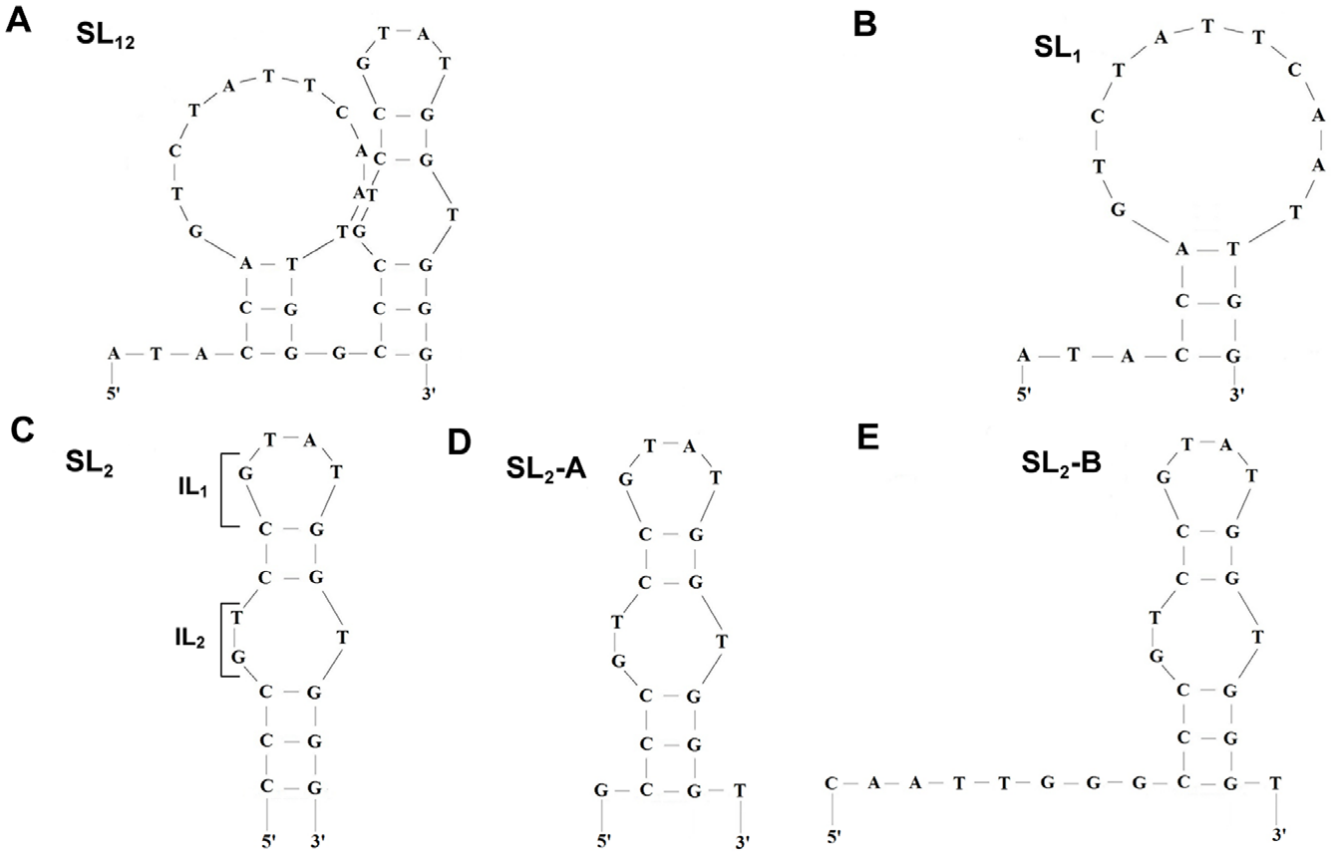
Schematic representation of the secondary structures of various truncated aptamers as predicted by the mfold program. (A) SL_12_ indicates the presence of SL_1_ and SL_2_ regions together, (B) SL_1_ indicates the presence of SL_1_ region only and (C) SL_2_ indicates the presence of SL_2_ region only. (D) SL_2_-A and (E) SL_2_-B correspond to secondary structures formed after addition of nucleotides to SL_2_ region.

To better understand the significance of stem-loop (SL) regions towards binding affinity, we conducted a few truncations on the SL regions. By truncating SL_3_ at the 3′ end together with 3′-termini hanging nucleotides and the nucleotides between SL_2_ and SL_3_, we obtained SL_12_ (Figure 3A and Table 1) and it exhibited a K_d_ value of 5 nM, a striking 24-fold increase in the binding affinity compared with the original VEa5 aptamer. Further truncation of SL_2_ to yield SL_1_ (Figure 3B and Table 1), however, resulted in complete loss of binding activity towards VEGF_165_. These two modifications pointed to the indispensable role of SL_2_ in the binding process. The increase in the binding affinity could be due to the deletion of non-binding nucleotides that hampers the binding process and hence, their removal may lead to more desired secondary conformation in the truncated aptamer required for binding to the HBD of VEGF_165_ protein [28].

We next truncated SL_1_ and left with only the SL_2_ sequence for VEGF_165_ binding analysis, and surprisingly we obtained lower binding affinity. With K_d_ value of 49 nM (Figure 3C and Table 1), the binding affinity of the SL_2_ sequence was approximately 10 times higher than the K_d_ of SL_12_. This prompted us to further investigate the role of the additional sequence next to the 3′ and 5′ ends of the SL_2_ region. By adding a single nucleotide at both 5′ and 3′-ends of the SL_2_ aptamer (SL_2_-A, Figure 3D), we were able to lower the K_d_ value by almost 5-fold (K_d_ = 10 nM, Table 1) compared to the SL_2_ aptamer. Further addition of nucleotides to 3′-end did not yield any improvement in the binding affinity. However, adding another 7 nucleotides at 5′-end of the SL_2_ aptamer (SL_2_-B, Figure 3E) showed further enhancement in the binding affinity (K_d_ = 0.5 nM, Table 1), and this represented a 90-fold increase compared with the SL_2_ aptamer, and more than 200-fold increase compared with the original VEa5 aptamer and 10-fold increase compared with the dimerized VEa5 aptamer [36]. A possible explanation is that the addition of nucleotides to the 5′-end provides more conformational stability to the aptamer which helps in improving the binding competence of the SL_2_-B sequence [38]. We also attempted to truncate the internal loops of SL_2_ (IL_1_ and IL_2_, Figure 2) but the removal of either loops reduced the binding affinity. Therefore, SL_2_-B is thought to be the minimal sequence required in VEa5 aptamer to provide high binding affinity to HBD of VEGF_165_.

To address the selectivity of the truncated SL_2_-B aptamer to VEGF_165_ binding, VEGF_121_ was selected for the binding comparison. VEGF_121_ is another spliced form of VEGF mRNA that constitutes only the receptor-binding domain (RBD) but is devoid of HBD. HBD assists in the binding of VEGF protein to the heparin sulfate (HS) and heparin sulfate proteoglycans (HSPGs) present on the extracellular matrix of the cell membrane [32]. This enhances the interaction of VEGF with its receptors (VEGFR-1/Flt-1 and VEGFR-2/KDR) and the specific co-receptor neuropilins, triggering the cellular angiogenic response in malignant cells [34].

**Figure 4:**
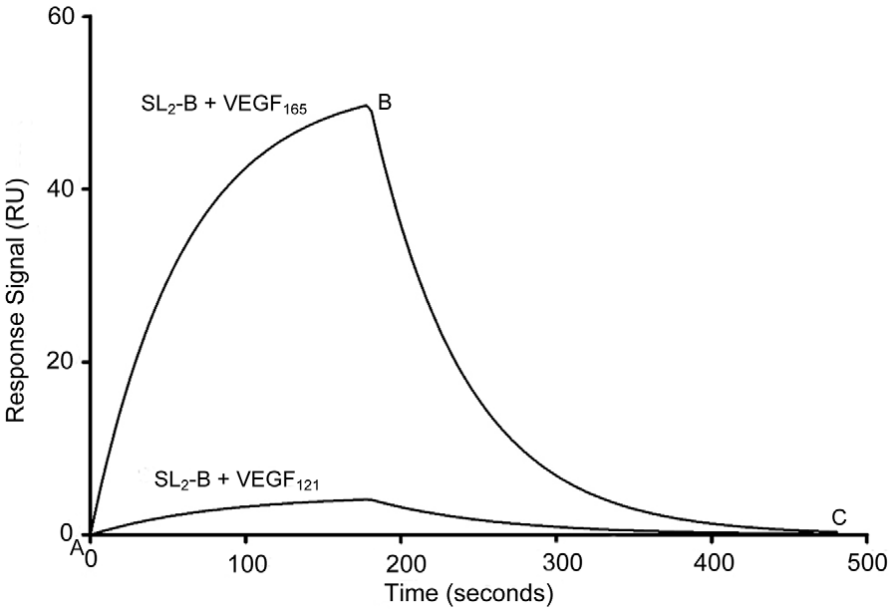
Sensorgrams demonstrating interaction of SL_2_-B aptamer with immobilized VEGF_165_ and VEGF_121_ proteins using SPR spectroscopy. Point A to B corresponds to the association phase and point B to C corresponds to the dissociation phase in the sensorgrams. Shown here is SL_2_-B aptamer binding with VEGF_165_ protein (K_d_ = 0.50±0.32 nM) and VEGF_121_ protein (K_d_ = 10.2±1.89 µM).

Compared with VEGF_165_, the binding of VEGF_121_ to the membrane receptors is not strong, making it not potent to generate intense angiogenic signal. Using SL_2_-B for binding analysis with VEGF_121_ under same buffer condition (Figure 4), substantial reduction in the response signal and minimal binding (K_d_ = 10.2 µM) was observed compared to VEGF_165_. This confirms the binding selectivity of the truncated SL_2_-B aptamer on HBD of the VEGF_165_ protein.

### *In vitro* cellular binding analysis of SL_2_-B aptamer-VEGF complex

Compared to techniques such as nitrocellulose membrane filtration or capillary electrophoresis, cell-based technique, such as flow cytometry, isolates aptamer sequences that have the ability to bind to their target with high affinity and specificity in a more physiologically relevant environment [39], [40]. Thus, it has been considered as a more direct strategy for studying the binding of aptamers to their targets on cellular surface.

**Figure 5:**
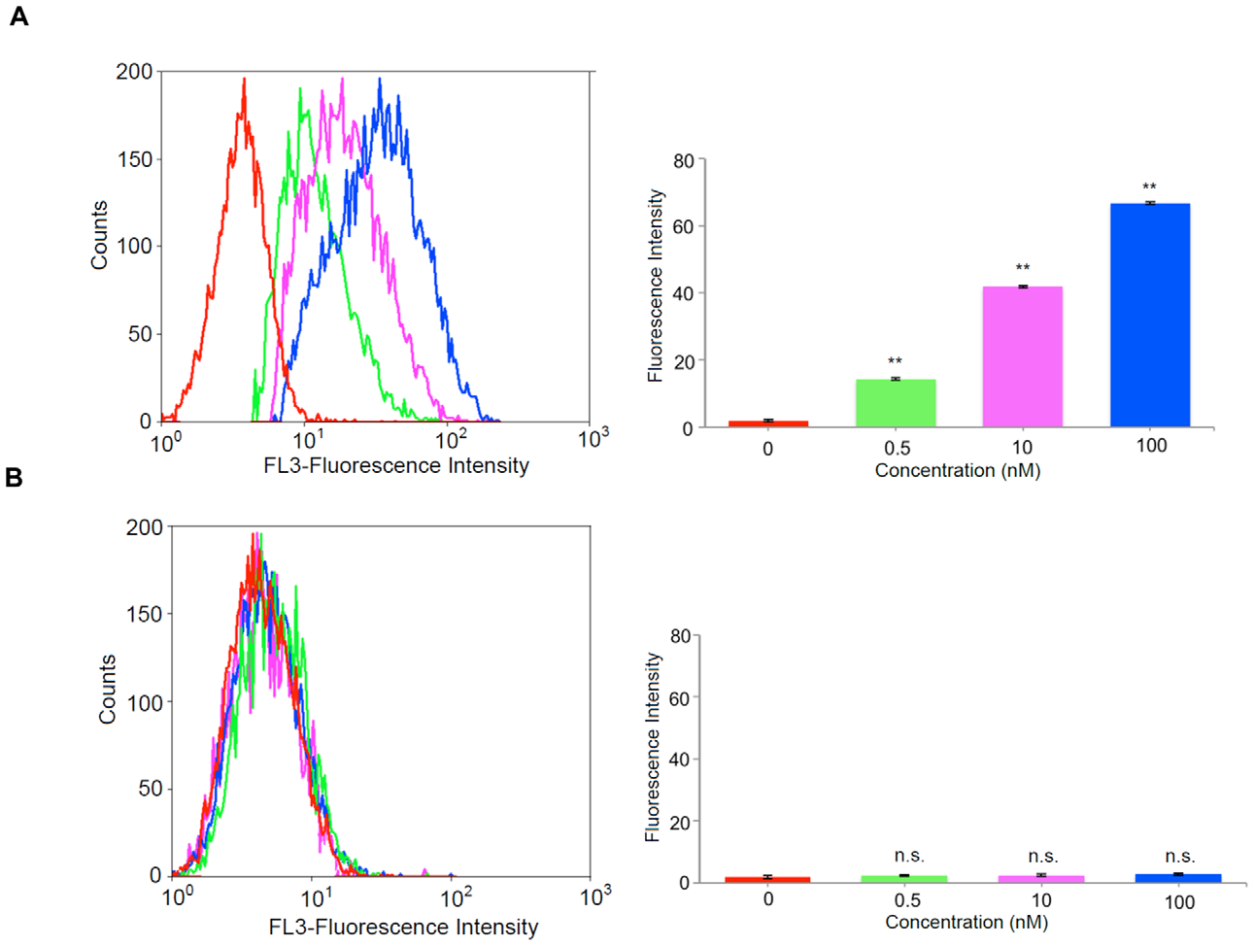
Representative flow cytometry profiles and quantitative analysis of flow cytometry results in (A) HT-29 cells and (B) MRC-5 cells after SL_2_-B aptamer at different concentrations (red – 0 nM (negative control), green – 0.5 nM, pink – 10 nM, blue – 100 nM), **Significant difference compared with the negative control (p-value<0.001), n.s. – not significant.

**Figure 6:**
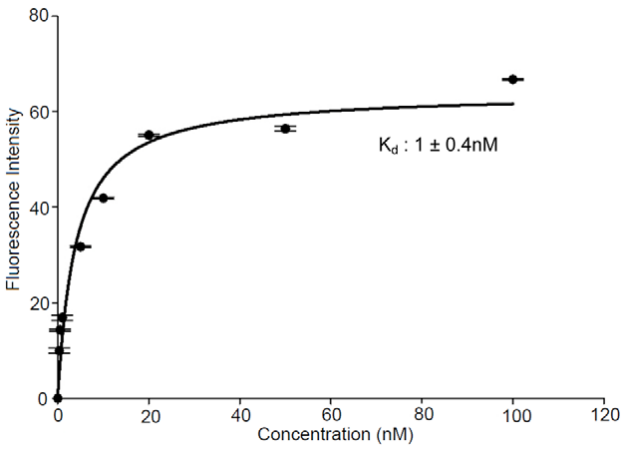
Binding curve of SL_2_-B aptamer with HT-29 cells. Cells were incubated with different SL_2_-B aptamer concentrations ranging from 0 to 100 nM. The fluorescence intensity originating from the scrambled sequence at each concentration was subtracted from the fluorescence intensity of corresponding SL_2_-B aptamer. The actual fluorescence intensity was fitted into SigmaPlot software to determine the K_d_.

To validate the increase in the binding affinity of the truncated SL_2_-B aptamer and to determine its target specificity at cellular level, we exposed the fluorescent-labeled SL_2_-B sequence directly on the HT-29 colorectal cancer cells and normal MRC-5 fibroblast cells (control) under 1% O_2_ hypoxia condition by flow cytometry analysis. As shown in Figure 5A, a significant peak shift (increase in the fluorescent signal) was observed for SL_2_-B aptamer at different concentrations, compared to control sample (only cells) in response to hypoxia conditions in HT-29 cells (p-value<0.001). The percentage of fluorescent-labeled cells increased with the increase in the concentration of the SL_2_-B aptamer, indicating the binding of the SL_2_-B aptamer to the cell surface. In contrast, when using normal MRC-5 fibroblast cells, no enhancement in the fluorescent signal was observed after treatment with different SL_2_-B aptamer concentrations (Figure 5B, not significant (n.s.) compared to negative control). Since the VEGF protein is overexpressed in HT-29 cells but not the normal MRC-5 cells, the result showed that SL_2_-B can specifically form aptamer-VEGF complex on the HT-29 cell membrane. The K_d_ value of the SL_2_-B towards VEGF was further evaluated using flow cytometry data (Figure 6). The K_d_ value for the SL_2_-B aptamer to the cell surface was found to be 1.0 nM, which is close to the K_d_ value determined by SPR technique (Figure 6). Repeating the same experiment at normoxia condition (i.e. 21% O_2_) did not yield any observable binding of SL_2_-B aptamer to the cells. The result is in agreement with the fact that the hypoxia condition enhances production of VEGF protein in HT-29 cells [41]–[43].

Hypoxia is one of the crucial physiological factors that exert profound impact on the metabolism, invasion and tumor progression, thereby affecting many oncogenic pathways. Inadequate level of cellular oxygen leads to aggressive phenotypic changes in the tumor cells, and is primarily responsible for their resistance against the therapies and poor prognosis [44]. Exposure to hypoxia milieu upregulates the expression of master regulator Hypoxia-Inducible Factor 1 gene (HIF-1 gene) in the solid tumors, which switch on the transcription of several downstream genes, in particular, VEGF [45]. The expression of VEGF protein and its two tyrosine kinase receptors – VEGFR-1/Flt-1 and VEGFR-2/KDR is induced by the transcription and stabilization of VEGF mRNA in response to hypoxia, resulting in increase rate of vascularization in tumor [46]–[48].

**Figure 7:**
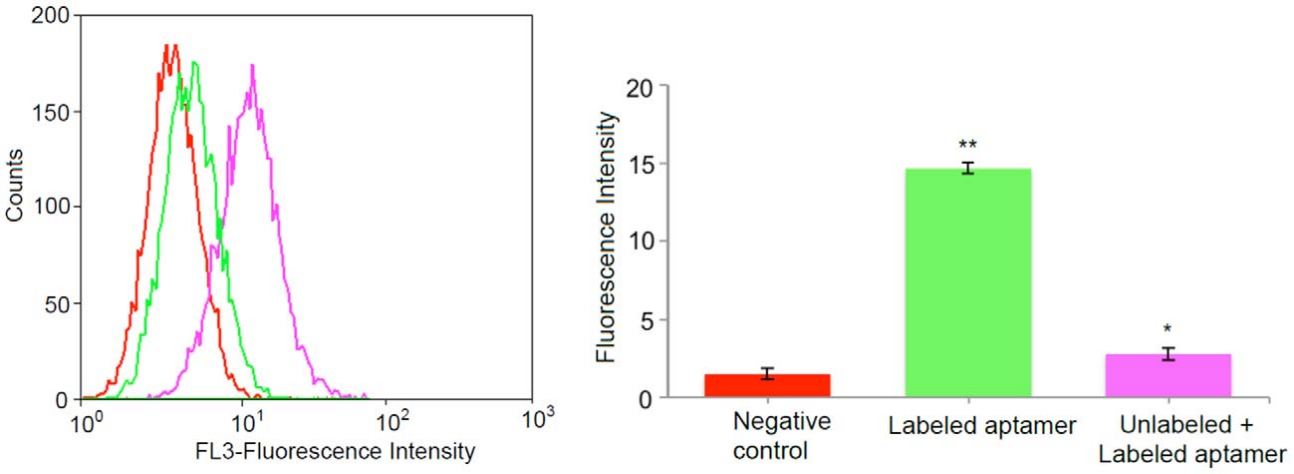
Flow cytometry histogram showing binding competition between the labeled and unlabeled SL_2_-B aptamer in HT-29 cells and quantitative analysis of flow cytometry result. Red – 0 nM (negative control), green – aptamer binding without excess of unlabeled SL_2_-B aptamer (0.5 nM); pink – aptamer binding in excess of unlabeled SL_2_-B aptamer (unlabeled aptamer – 10 nM, labeled aptamer – 0.5 nM). *Significant difference from the negative control sample at p-value<0.05; **Significant difference from the negative control sample at p-value<0.001.

The binding ability of SL_2_-B aptamer for VEGF protein in HT-29 cells was evaluated by competing 5′-PE-texas red labeled SL_2_-B sequence against the unlabeled SL_2_-B sequence. As shown in Figure 7, the presence of 20-fold excess of unlabeled aptamer significantly decreased the fluorescent signal from the PE-texas red labeled aptamer (p-value<0.05). This result suggests that both the labeled and unlabeled SL_2_-B aptamer binds to the same target, the VEGF protein on HT-29 cell membrane.

### *In vitro* fluorescence imaging of SL_2_-B aptamer-VEGF complex

**Figure 8:**
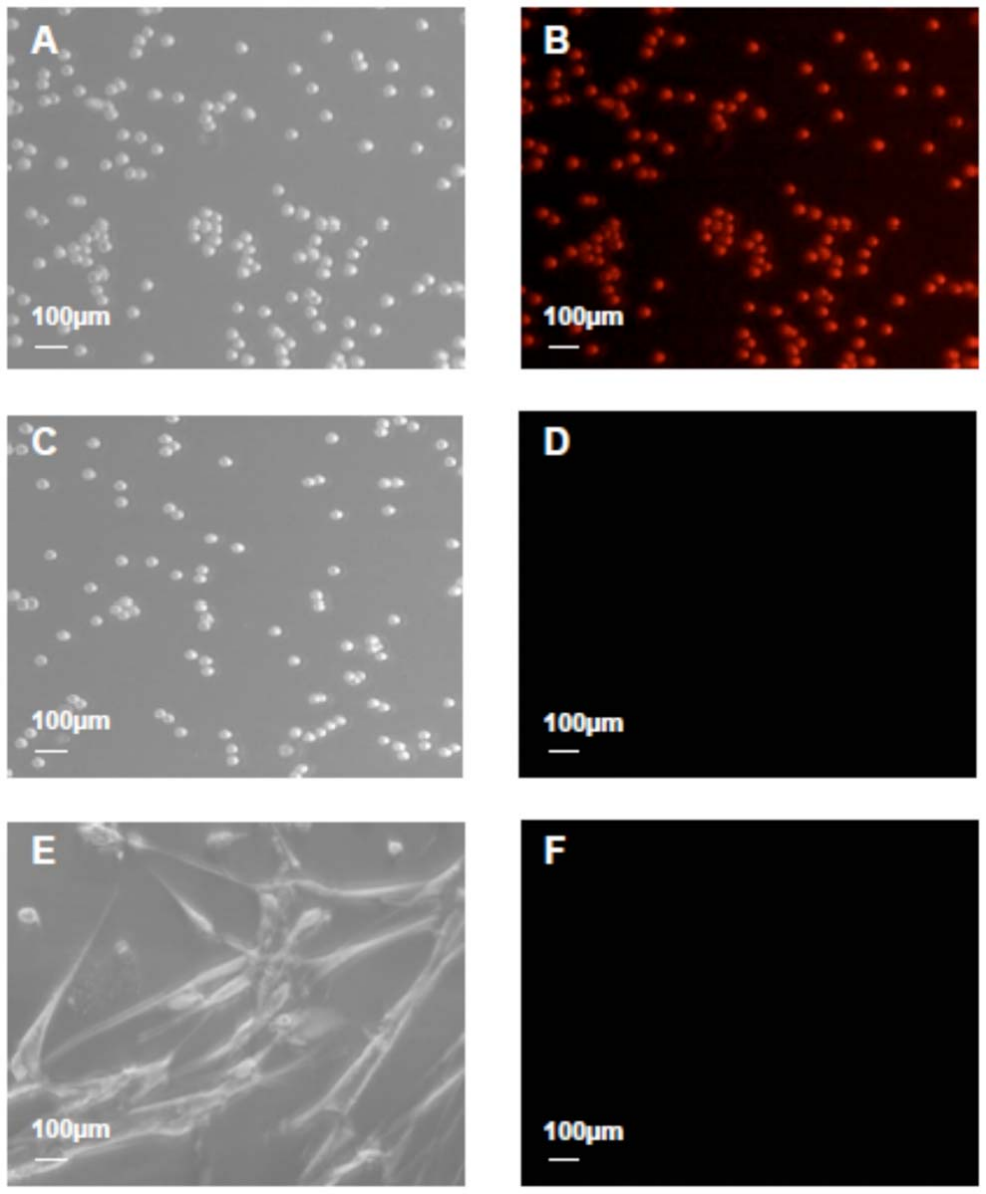
Binding of PE-texas red-labeled aptamers (SL_2_-B and scrambled sequence) to HT-29 cells and normal MRC-5 fibroblast cells under hypoxia condition. (A) & (C) Bright field images of HT-29 cells after exposing to the SL_2_-B and scrambled sequence respectively. (B) & (D) Corresponding fluorescence microscopy images of the (A) & (C) bright field images. (E) & (F) Bright field and fluorescence microscopy images of MRC-5 cells after exposing to the SL_2_-B sequence.

The targeting ability and specificity of the high affinity SL_2_-B aptamer was further investigated and imaged using live colorectal cancer HT-29 cells and normal MRC-5 fibroblast cells (control) at the same hypoxia conditions. By comparing the bright field and the fluorescent images, we observed red fluorescence on HT-29 cells after exposing to PE-texas red labeled SL_2_-B aptamer (Figure 8A & B), but no detectable fluorescence was observed with PE-texas red labeled scrambled sequence (Figure 8C & D). This confirms the specific binding of SL_2_-B aptamer to VEGF_165_ protein in cancer cells. For MRC-5 fibroblasts, minimal fluorescence was also observed after exposing to the same SL_2_-B aptamer (Figure 8E & F). This further demonstrates the specificity and targeting ability of the SL_2_-B aptamer to cancer cells.

### Conclusions

To summarize, this work attempted to identify in the key binding aptamer sequences by truncating based on the stem-loop structure of the original VEa5 aptamer. From the results, we can conclude that the SL_2_ sequence is important for the binding to HBD of the VEGF_165_ protein, and the SL_2_-B aptamer binds HBD of VEGF_165_ protein strongly and selectively. This newly obtained SL_2_-B aptamer sequence can potentially be useful in oligomer-based cancer therapeutic and diagnostic applications, though further studies are required for better understanding of the SL_2_-B aptamer sequence and to elucidate its binding mechanism with HBD of VEGF_165_ protein. Furthermore, the current stem-loop modification approaches can be useful in identifying target domains, getting rid of excessive unnecessary nucleotides, and eventually lowering the cost of aptamer synthesis.
